# Potential of Novel Magnesium Nanomaterials to Manage Bacterial Spot Disease of Tomato in Greenhouse and Field Conditions

**DOI:** 10.3390/plants12091832

**Published:** 2023-04-29

**Authors:** Ying-Yu Liao, Jorge Pereira, Ziyang Huang, Qiurong Fan, Swadeshmukul Santra, Jason C. White, Roberto De La Torre-Roche, Susannah Da Silva, Gary E. Vallad, Joshua H. Freeman, Jeffrey B. Jones, Mathews L. Paret

**Affiliations:** 1 Department of Plant Pathology, University of Florida, Gainesville, FL 32611, USA; 2 North Florida Research and Education Center, University of Florida, Quincy, FL 32351, USA; 3 Department of Chemistry, University of Central Florida, Orlando, FL 32816, USA; 4 NanoScience Technology Center, University of Central Florida, Orlando, FL 32826, USA; 5 Burnett School of Biomedical Sciences, University of Central Florida, Orlando, FL 32816, USA; 6 Department of Analytical Chemistry, The Connecticut Agricultural Experiment Station, New Haven, CT 06511, USA; 7 Gulf Coast Research and Education Center, University of Florida, Wimauma, FL 33598, USA

**Keywords:** nanoparticles, antimicrobial, crystallinity, *Xanthomonas perforans*, crop protection

## Abstract

Bacterial spot of tomato is among the most economically relevant diseases affecting tomato plants globally. In previous studies, non-formulated magnesium oxide nanoparticles (nano-MgOs) significantly reduced the disease severity in greenhouse and field conditions. However, the aggregation of nano-MgO in liquid suspension makes it challenging to use in field applications. Therefore, we formulated two novel MgO nanomaterials (SgMg #3 and SgMg #2.5) and one MgOH_2_ nanomaterial (SgMc) and evaluated their physical characteristics, antibacterial properties, and disease reduction abilities. Among the three Mg nanomaterials, SgMc showed the highest efficacy against copper-tolerant strains of *Xanthomonas perforans* in vitro, and provided disease reduction in the greenhouse experiments compared with commercial Cu bactericide and an untreated control. However, SgMc was not consistently effective in field conditions. To determine the cause of its inconsistent efficacy in different environments, we monitored particle size, zeta potential, morphology, and crystallinity for all three formulated materials and nano-MgOs. The MgO particle size was determined by the scanning electron microscopy (SEM) and dynamic light scattering (DLS) techniques. An X-ray diffraction (XRD) study confirmed a change in the crystallinity of MgO from a periclase to an Mg(OH)_2_ brucite crystal structure. As a result, the bactericidal activity correlated with the high crystallinity present in nano-MgOs and SgMc, while the inconsistent antimicrobial potency of SgMg #3 and SgMg #2.5 might have been related to loss of crystallinity. Future studies are needed to determine which specific variables impair the performance of these nanomaterials in the field compared to under greenhouse conditions. Although SgMc did not lead to significant disease severity reduction in the field, it still has the potential to act as an alternative to Cu against bacterial spot disease in tomato transplant production.

## 1. Introduction

Bacterial spot of tomato, caused by four *Xanthomonas* species [1], is one of the most devastating bacterial diseases with high economic relevance in Florida. Florida is one of the major suppliers of tomato transplant seedlings for fresh-market tomato production in the northeast and central USA [2]. Pathogen-free certified seeds and disease-free transplants are recommended as primary cultural management strategies for managing bacterial spot of tomato [3]. However, given the difficulty of guaranteeing pathogen-free seeds and the need to use overhead irrigation, maintaining disease-free transplants is a challenge [4]. Disease management approaches in transplant facilities use a combination of cultural practices and chemical sprays [4,5]. Field management relies on a multipronged strategy, including cultural methods for the purpose of, for example, eliminating volunteer plants, minimizing crop residue, and eradicating weeds [4] and chemical sprays, for which growers rely heavily on Cu bactericides [5]. However, continuous and heavy use of Cu bactericides may lead to challenges in environmental safety due to metal accumulation in the soil [6].

Considering the negative aspects of Cu use and its accumulation over time, identifying sustainable and effective alternative management strategies is critical. In a recent study, Abrahamian et al. (2019) [5] evaluated different alternatives to Cu bactericides in the transplant house. They determined that acibenzolar-S-methyl (ASM), quinoxyfen, oxysilver nitrate, and pentasilver hexaox-oiodate significantly reduced disease compared to the controls. On the other hand, under field production, ASM can negatively impact fruit yield [7]. Florida tomato growers continue to use a combination of Cu bactericides, ASM, bio-fungicides, and cultural practices [4,8,9]. Since Cu-tolerant strains have evolved, commercial Cu bactericides have become ineffective. Currently there are no effective alternative chemical control strategies utilizing bactericides [4,10]. In addition, in 2017, the EPA suggested that Cu bactericides be reduced based on ecological risks for non-target organisms exposed to field runoff containing Cu (EPA-HQ-OPP-2010-0212). Therefore, finding an effective alternative to Cu is a long-term goal. Nanotechnology is a growing field in agriculture for plant disease management. In a previous study, we demonstrated the effectiveness of nano-MgO against a Cu-tolerant strain of *X. perforans* [11,12], and demonstrated that nano-MgO has a high level of bactericidal activity against the bacterial strain. In greenhouse and field experiments, bacterial spot disease severity was significantly reduced by nano-MgO applications at 200 µg/mL compared to the untreated water control (UT), whereas the grower-standard treatment, copper hydroxide + ethylene-bis-dithiocarbamate (Cu-EBDC), was not significantly different from the control with untreated water (*p* = 0.05). In order to determine whether nanoparticles accumulated in fruit, inductively coupled plasma mass spectrometry was used to analyze fruits from nano-MgO-treated plots. The levels of Mg, Cu, Ca, K, Mn, P, and S were not significantly altered compared to the untreated water control. As a result, if commercialized, nano-MgO could potentially be applied as an alternative to Cu in an integrated pest management (IPM) program to reduce bacterial spot disease. In this scenario, selective pressure for the development of Cu-tolerant *X. perforans* in the field would also be reduced [13]. However, the nano-MgO material is non-formulated (crude grade); it is an uncharacterized, unevenly dispersed, and heterogeneous suspension. Because the nanoparticles were likely aggregated, the antibacterial agent might have actually limited contact with the pathogen. This may explain why there was variation in the greenhouse and field experiments, with the higher concentration tending to have a greater standard deviation than the lower concentration in the prior study [12]. This phenomenon of aggregation fit the observation in the previous study by Sawai et al. (2003) that the antibacterial ability was contact-dependent between the MgO nanoparticles and the target bacteria [14].

For Nano-MgO, one of the concerns surrounding other metal oxide nanoparticles such as TiO_2_, ZnO, CeO_2_, MeO, SiO_2_, and Fe_2_O_3_ was shared, in that it tended to aggregate in different aquatic environments [15,16,17]. In general, commercially available metal oxide nanoparticles are in powder form rather than in suspension. Some nanoparticles including TiO_2_ would aggregate in water, and it has been difficult to break the agglomerates into primary nanoparticles [18,19]. With the formation of hematite after long-term storage, the aggregation of nanoparticles could not be broken by ultrasound nor by chemical dispersants in some situations [16]. The aggregates made the chemical application challenging in the field. This aggregating phenomenon highlights the importance of developing MgO formulations as more homogeneous suspensions, possibly through the addition of surface coatings, functional groups, and/or polymers to control particle dissolution. This type of formulated Mg nanoparticle would likely result in an effective antibacterial agent that could be used as an alternative to Cu bactericide. Another interesting observation about MgO as a nanoparticle is that upon exposure to humid conditions or water, the MgO surface is converted into Mg(OH)_2_ [20,21]. Therefore, it is important to analyze whether a nano Mg(OH)_2_ would provide similar control as that of a nano-MgO.

In this study, we hypothesized that a formulated Mg nanomaterial would have improved antibacterial properties in vitro, and, therefore, would provide a method for effectively controlling bacterial spot of tomato in the greenhouse and in field conditions when compared to commercial micron-sized Cu. Therefore, we evaluated (i) the transformation of Mg species (MgO and MgOH_2_) upon formulation with citric acid, and (ii) the efficacy of formulated Mg nanomaterials against Cu-tolerant *X. perforans* in vitro, in the greenhouse as well as in field conditions.

## 2. Results

### 2.1. Material Characterization

In order to characterize each nanomaterial, we evaluated the effect of increasing the citrate concentration in SgMg on its colloidal property, and compared the results with the nonformulated Mg nanoparticle (nano-MgO). The results are summarized in Table 1. DLS studies reveal that both nonformulated nano-MgO and the formulated SgMg #3, which had a lower citrate concentration, exhibited large particulates in suspension. These large particulates were suspected to be the agglomerates of the primary particles, accounting for their limited colloidal stability in the suspension. However, SgMg #2.5, with a higher citrate concentration, showed much smaller particle sizes, with relatively narrow size distribution in comparison to the SgMg #3. This phenomenon suggests that there is a threshold concentration of citrate, above which smaller-sized primary particles are stabilized in solution. In addition, a significant change in ζ value from +11.4 mV to −43.3 mV confirmed that both SgMg formulations had citrate binding on their particle surfaces. The higher magnitude of the ζ value, either +ve or −ve, would indicate better dispersibility of particles and, thus, colloidal stability (via Coulombic interaction, also known as electrostatic interactions) in comparison to their nonformulated counterparts with lower ζ values [22,23,24]. Last but not least, it has been documented that particle instability can cause increases in particle size, where large particles in a suspension grow to bigger sizes through the dissolution of smaller-sized particles, a well-established colloidal phenomenon known as Ostwald ripening [25,26]. Therefore, a well-passivated particle surface with a greater value of c is expected to possess a longer shelf-life due to minimization of the Ostwald ripening.

**Figure 1:**
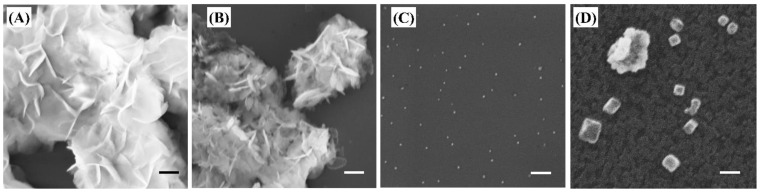
Characterization of formulated Mg nanomaterials. SEM images of original nano-MgO dispersed in DI water, followed by drying (**A**); the formulated Mg materials SgMg #3 (**B**) and SgMg #2.5 (**C**); and a representative image of SgMc (**D**), as reported by Huang et al. [27]. The scale bars were set to 200 nm.

After using DLS to determine the particle size in the suspension, SEM was utilized to characterize the morphologies and sizes of the particles in the dry state. As a result, SgMg #2.5 showed fully dispersed primary particles (~20 nm; Figure 1C). However, SgMg#3, which had a lower citrate concentration, did not produce nano-sized particles. Instead, SgMg #3 resulted in micron-sized particulates with nano-sized surface features (Figure 1B). These features had laminar shapes, approximately 250 nm in diameter and 20 nm in thickness, respectively. Figure 1A reveals that nonformulated nano-MgO aggregated in DI water and formed micron-sized particulates with irregular surfaces as well. In combination with the DLS measurements, the SEM results suggest that there were colloidal aggregation in the nano MgO formulations. Given that lower nano-MgO exhibited better antimicrobial efficacy [11], our findings justify the need for producing nanoformulations of MgO using a suitable capping agent, such as citric acid.

**Figure 2:**
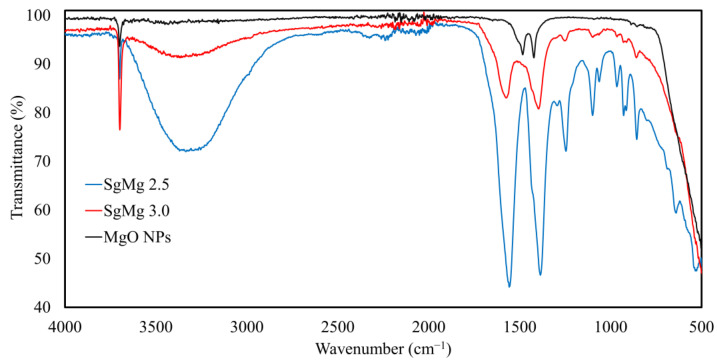
Fourier-transformed infrared (FTIR) spectra of the formulated and unformulated MgO nanomaterials. The formulated materials’ (SgMg #3 and SgMg #2.5) spectra demonstrate the characteristic peaks of citrate, whereas the unformulated MgO NPs display the absence of the characteristic functional groups.

Therefore, we further characterized the property of each nanomaterial. Figure 2 displays the FTIR spectra of the purified samples. The spectra revealed that the characteristic stretching frequencies for hydroxyl, carboxylate asymmetric, and carboxyl symmetric of citrate appeared at 3800 cm^−1^, 1550 cm^−1^, and 1383 cm^−1^, respectively [27,28]. This result also implies that citrate is chemically bound to the nano-MgO surface. It was noted that the FTIR absorption intensity drastically increased for the SgMg #2.5 surface in comparison to that of SgMg #3, confirming the significantly higher amount of citrate molecules on the particle. In other words, smaller particles (SgMg #2.5) can retain greater amounts of citrate on the surface due to their high surface-to-volume ratio compared to larger particles (SgMg #3).

**Figure 3:**
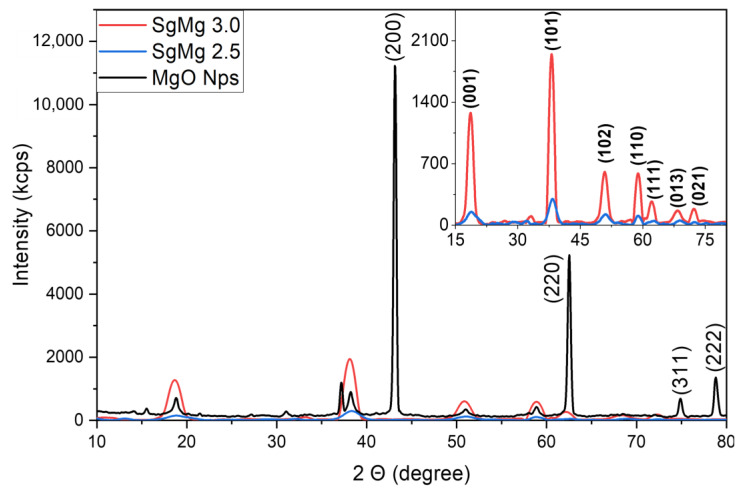
XRD spectra of the formulated and unformulated nano-MgOs. Peak assignment was based on previous studies using the JCPDS database. Unformulated MgO displayed the characteristic periclase peak patterns, while formulated materials (SgMg) displayed brucite crystalline structures. Moreover, the formulated MgO showed a markedly lower intensity compared to the nano-MgO, which suggests significantly lower crystallinity. The top right inset is a magnification of SgMg #3 and SgMg #2.5 diffractograms.

After confirming the size and citrate concentration in both SgMg nanomaterials, we used XRD (Figure 3) to demonstrate that the formulated nano-MgO had a different crystalline structure (brucite; JCPDS 84-2163) [27,29] than that of the original nonformulated nano-MgO (periclase; JCPDS 87-0653) [30,31,32,33]. The XRD data suggest that the citrate ions facilitated the transformation of the crystalline structure from periclase to brucite. This transition would confirm that the SgMg materials are primarily Mg(OH)_2_ species instead of nano-MgO. Moreover, the diffraction intensities of SgMg #3 and SgMg #2.5 were substantially lower compared to the nonformulated nano-MgO, suggesting a loss of crystallinity in SgMg. Besides SgMg#2.5 and #SgMg 3, another Mg nanomaterial, SgMc, was also included in this study. The physical properties of SgMc have been reported previously [27].

Huang et al. (2018) [22] reported that SgMc exhibited a hydrodynamic diameter of 280 nm and a ζ of −16.9 mV. Additionally, the average diameter of the primary particles was measured to be approximately 100 nm based on SEM. Moreover, XRD studies demonstrated that SgMc has a brucite crystalline lattice, which has been confirmed to be mostly composed of Mg(OH)_2_. In general, the physical properties of SgMc are similar to those of SgMg #2.5, but SgMc exhibits a higher-intensity reflection in the XRD spectra, denoting higher crystallinity.

### 2.2. Antibacterial Activity of Formulated Mg Nanomaterials In Vitro

**Figure 4:**
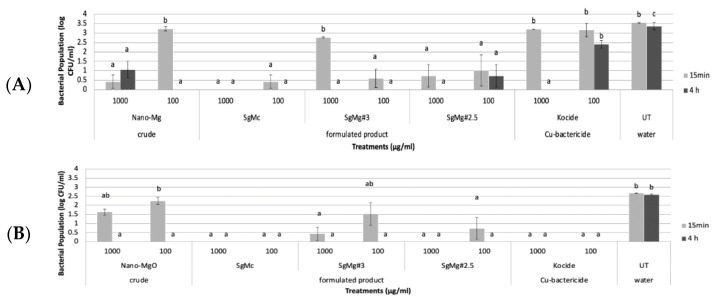
In vitro effects of formulated Mg nanomaterials (SgMc, SgMg #3, SgMg #2.5) at 1000 and 100 µg/mL on bacterial growth over time (15 min and 4 h). The materials were compared to copper treatments (Kocide^®^ 3000) and crude nano-MgO at 1000 (3.3 g/L) and 100 µg/mL (0.334 g/L). (**A**) The treatments were effective against Cu-tolerant strain XpGEV485 and (**B**) against Cu-sensitive strain Xp91-118. Numbers with different letters in the same column were significantly different (*p* = 0.05) based on Student–Newman–Keuls statistical analysis using the IBM^®^ SPSS^®^ program.

The antibacterial activity of three formulated Mg nanomaterials (SgMc, SgMg #3, and SgMg #2.5) was evaluated compared with the non-formulated nano-MgO used in this study. In the first set of in vitro experiments, all three formulated Mg nanomaterials, as low as 100 µg/mL, showed significant antimicrobial activity against both Cu tolerant and Cu sensitive *X. perforans* after 15 min (Figure 4A,B). The non-formulated nano-MgO particle, as low as 100 µg/mL, showed significant antimicrobial activity against both Cu-tolerant and Cu-sensitive *X. perforans* after 4 h (Figure 4A,B). Cu bactericide Kocide 3000 was effective at both concentrations after 15 min against Cu-sensitive *X. perforans*, and at 1000 µg/mL after 4 h against Cu-tolerant *X. perforans* (Figure 4A,B).

**Figure 5:**
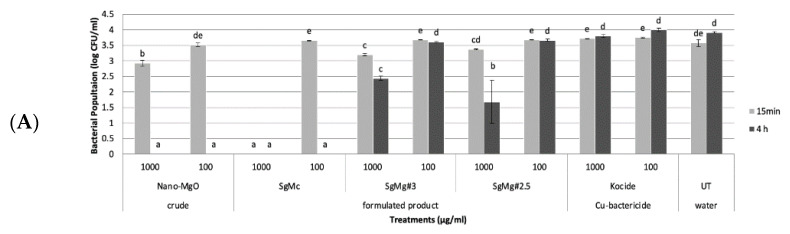

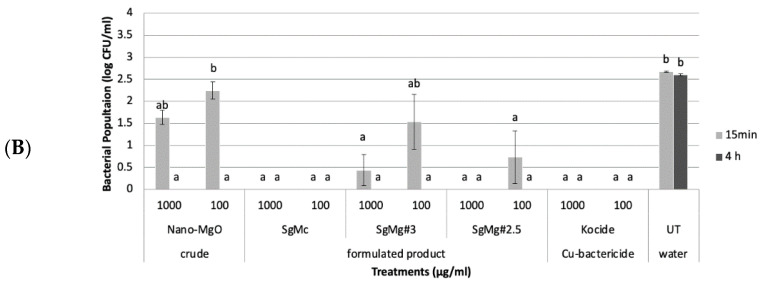
In vitro effect of formulated Mg nanomaterials (SgMc, SgMg #3, and SgMg #2.5) on bacterial growth over time (15 min and 4 h). The materials were compared to copper treatments (Kocide^®^ 3000) and crude nano-MgO at 1000 (3.3 g/L) and 100 µg/mL (0.334 g/L). (**A**) The treatments were against Cu-tolerant strain XpGEV485. (**B**) The treatments were against Cu-sensitive strain Xp91-118. Numbers with different letters in the same column showed significant differences (*p* = 0.05) based on Student–Newman–Keuls statistical analysis using the IBM^®^ SPSS^®^ program.

In the second set of in vitro experiments, the formulated Mg nanomaterial, SgMc, was consistently effective against both Cu-tolerant and Cu-sensitive *X. perforans* at 100 µg/mL after 15 min (Figure 5A,B). The two formulated Mg nanomaterials, SgMg #3 and SgMg #2.5, had significant antibacterial activity at 1000 µg/mL after 4 h (Figure 5A,B). As for non-formulated nano-MgO, it consistently had significant antimicrobial activity against both Cu-tolerant and Cu-sensitive *X. perforans* at concentrations as low as 100 µg/mL after 4 h (Figure 5A,B). Cu bactericide was effective against Cu-sensitive *X. perforans* at as low as 100 µg/mL after 15 min (Figure 5A), but was not effective against Cu-tolerant *X. perforans* compared to the untreated water control (Figure 5B).

**Figure 6:**
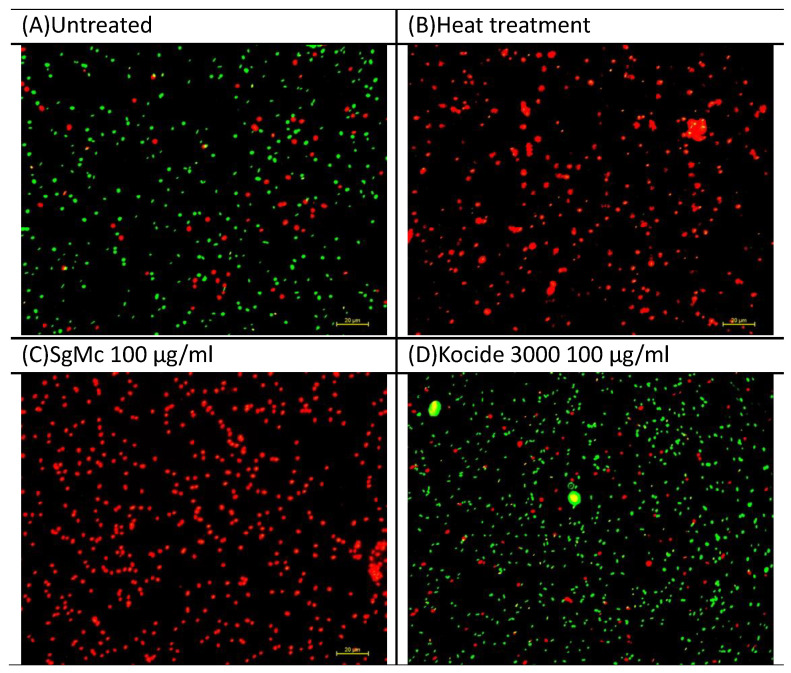
Viability assay of Cu-tolerant *X. perforans* GEV485 treated with 100 µg/mL formulated Mg nanomaterial (SgMc). Untreated and heat-treated samples, as well as those treated with the Cu bactericide Kocide^®^ 3000, were used as the controls. For cells stained with the LIVE/DEAD^®^ BacLight™ Bacterial Viability Kit, green indicates live cells and red indicate dead cells. Micrographs were taken on a Nikon Eclipse Ti inverted microscope (Nikon, Melville, NY, USA) at ×40 fluorescent optics using NIS-Elements imaging software (ver. 3.0; Nikon).

Since the formulated Mg nanomaterial SgMc consistently had significant antibacterial activity against Cu-tolerant *X. perforans*, SgMc was tested in a viability assay. The bactericidal activity was confirmed by the viability assay (Figure 6A–D). All bacteria were killed (100% mortility) (red fluorescence) after treatment with 100 µg/mL SgMc (Figure 6C) for 4 h, similarly to the heat-treated positive control (Figure 6B), as indicated by all cells exhibiting red fluorescence. In comparison, Cu bactericide (Kocide 3000) (Figure 6C) had a mortality percentage of 19.3%, similar to the untreated water control (20%) (Figure 6A).

### 2.3. Comparison of the Efficacy of Formulated Mg Nanomaterials with Nano-MgO, Cu, and Cu-EBDC for the Management of Tomato Bacterial Spot in Greenhouse Conditions

**Figure 7:**
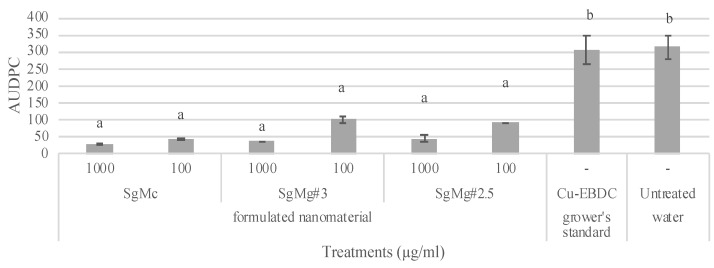
Greenhouse experiment conducted in the summer of 2017: bacterial spot disease severity was evaluated by the following treatments: 100 and 1000 µg/mL of formulated Mg nanomaterials (SgMc, SgMg #3, SgMg #2.5); and a combination of Kocide^®^ 3000 (2.1 g/L), Penncozeb^®^ 75DF (1.2 g/L) (Cu-EBDC), and sterile tap water. Error bars = standard error. Numbers with different letters in the same column showed significant differences. A *p*-value of 0.05 was used for the IBM^®^ SPSS^®^ SNK (Student–Newman–Keuls) statistical analysis.

In the first greenhouse experiment in Auburn, AL, plants treated with both concentrations (100 or 1000 µg/mL) of the formulated Mg nanomaterial treatments (SgMc, SgMg #3, and SgMg #2.5) showed significantly less disease severity compared to the grower’s standard, Cu-EBDC, and the untreated water control (*p* = 0.05) (Figure 7).

**Figure 8:**
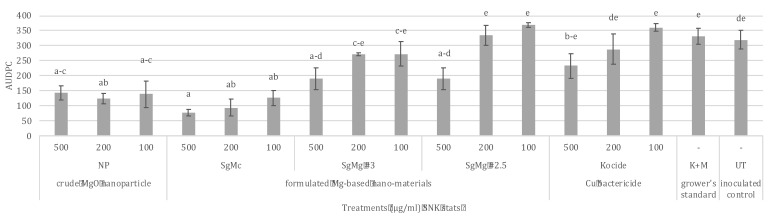
Greenhouse experiment conducted in the fall of 2017: bacterial spot disease severity was evaluated by the following treatments: 100, 200, and 500 µg/mL of formulated Mg nanomaterials (SgMc, SgMg #3, SgMg #2.5) and crude nano-MgO; and a combination of Kocide^®^ 3000 (2.1 g/L), Penncozeb^®^ 75DF (1.2 g/L) (K + M), and sterile tap water. Error bars = standard error. Numbers with different letters in the same column showed significant differences. A *p*-value of 0.05 was used for the IBM^®^ SPSS^®^ SNK (Student–Newman–Keuls) statistical analysis.

In the second greenhouse experiment in Gainesville, FL, plants treated with 3 concentrations (100, 200, 500 µg/mL) of SgMc and non-formulated nano-MgOs showed significantly less disease severity compared to Cu-EBDC and the untreated water control (*p* = 0.05) (Figure 8). Cu bactericide and the grower’s standard did not reduce disease severity compared to the untreated water control (Figure 8).

The first field experiments were conducted in spring 2018 in two locations: Quincy, FL, and Wimauma, FL, USA. None of the treatments significantly reduced the disease severity compared to the untreated water control (Table 2).

In the second set of field experiments, conducted during the fall of 2018 in Quincy (Table 3), none of the treatments led to significant differences in disease severity compared to the untreated water control. In the Wimauma trial, plants which received a concentration of either SgMc (1000 µg/mL or 200 µg/mL) or Cu-EBDC had significantly lower disease severity compared to the Cu bactericide Kocide 3000, but these values were not different from the untreated water control (Table 3). No phytotoxicity was observed for any of the treatments in any of the field experiments.

When the trial was repeated again in the spring of 2019 at Quincy, FL (Table 4), 100 µg/mL of the nano-MgO (20 nm) and SgMc significantly reduced disease severity compared to the untreated water control, whereas the grower’s standard, Cu-EBDC, did not significantly reduce disease in the field trials compared to the untreated water control (Table 4). In the trial conducted during the spring of 2019 in Wimauma, FL, 1000 µg/mL of both the nano-MgO (20 nm) and SgMc significantly reduced disease severity compared to the untreated water control, whereas the grower’s standard, Cu-EBDC, did not significantly reduce disease in the field trials compared to the untreated water control (Table 4). However, the Cu bactericide Kocide 3000 was significantly more effective compared to Cu-EBDC and the untreated water control.

## 3. Materials and Methods

### 3.1. Nanomaterial Formulation and Synthesis

In order to understand the effects of formulating MgO nanoparticles on their physicochemical and antimicrobial properties, nano-MgO (MgO, 99+%, 20 nm) was purchased in powder form from U.S. Research Nanomaterials, Inc. (Houston, TX, USA), and resuspended using a citric acid (C₆H₈O₇) solution. The formulation was stirred with a magnetic stirrer for 20 min before adjusting the pH to 10.8 with an aqueous 2.0 M sodium hydroxide solution. Two different concentrations of C_6_H_8_O_7_ were used to formulate the nano-MgO (SgMg #3 and SgMg #2.5). These nano-MgO formulation characteristics were compared to those of a nano Mg(OH)_2_ formulation (SgMc). The SgMc material was synthesized using Mg salt, a bottom-up synthesis approach, following a previously published method [27]. Briefly, MgCl_2_ and trisodium citrate dihydrate (Na_3_C_6_H_5_O_7_) were dissolved in deionized water under mechanical stirring. Subsequently, the pH of this solution was adjusted to 10.8 by dropwise addition of an aqueous 2.0 M sodium hydroxide solution. All formulations were adjusted to a concentration of 20,000 μg/mL of Mg using DI water. The Mg precursor of SgMc was switched from MgCl_2_ (crystalline solid) to Mg(NO_3_)_2_ (technical grade solution) to prepare the test materials to support the 2019 field trial.

### 3.2. Material Characterization

To determine the hydrodynamic size of the formulated Mg particles, the as-synthesized formulation was diluted in DI water and sonicated for 1 min to disperse particles using a bath sonicator. Subsequently, the hydrodynamic diameter, polydispersity index (PDI, the deviation from the mean diameter), and zeta potential were measured using a Zetasizer ZS90 (Malvern Panalytical) equipped with a He–Ne 632.8 nm and set at a 90° scattering angle. To ensure reproducibility, the experiments were conducted in duplicate. For all other material characterizations, the formulations were purified via the centrifugation method, operating at 12,000 rpm for 10 min. After the centrifugation, the supernatant was discarded and the pellet was redispersed in DI water. The purification process was repeated twice to remove the unbound citric acid molecules and the excess ions.

The primary particle size and morphology of the particles were assessed using the SEM technique. The preparation of the SEM sample involved the following steps. (i) The purified particle suspension was diluted to approximately 100 μg/mL of Mg; (ii) 1 drop of the diluted suspension was placed onto a clean silicon wafer; and (iii) the sample was dried for 24 h using a silica gel desiccator. The SEM images were acquired using the in-lens detector of a Zeiss Nvision 40, applying an acceleration voltage of 5 kV. Fourier-transformed infrared spectroscopy (FTIR) studies were performed in order to confirm the adsorption of citrate ions to the surfaces of the particles. The FTIR samples were prepared by freezing and lyophilizing the purified particle suspension. The FTIR spectra were collected using a Shimadzu IR Spirit equipped with a single-reflection attenuated total reflectance attachment (QATR-S). The crystallinity of the particles was determined using XRD of the above lyophilized powder with a PANalytical Empyrean XRD spectrometer. The XRD spectra were compared with the database of the Joint Committee on Powder Diffraction Standards (JCPDS).

### 3.3. Bacterial Strain and Storage

An *X. perforans* strain, GEV485 (Cu-tolerant), was isolated from tomato plants in Florida for use in this study. Bacterial cells from pure cultures of these strains were suspended in sterile 30% glycerol solution and stored at −80 °C. For each experiment, bacteria were streaked from the glycerol stock onto nutrient agar (NA) medium (BBL, Becton Dickinson and Co., Cockeysville, MD, USA), incubated at 28 °C, and transferred every 24 to 48 h until use for streaking on NA plates for the inoculum. Bacterial cells were collected from cultures grown on NA for 24 h and suspended in 0.01 M MgSO_4_, and the suspensions were adjusted to absorbance at A_600_ = 0.3 (approximately 5 × 10^8^ CFU/mL). The final concentrations of the bacterial suspensions were adjusted to 10^8^ CFU/mL. To assess the nonformulated nanoparticles, nano-MgO was suspended and sonicated in autoclaved deionized water, adjusted to 10,000 μg/mL, and used as a stock suspension.

### 3.4. In Vitro Assays Evaluating Direct Inhibition of the Growth of X. perforans

In order to evaluate the bactericidal activity of the formulated Mg nanomaterials in controlling bacterial spot disease of tomato, these materials were compared with non-formulated nano-MgO and Cu bactericide Kocide 3000 (a micron-sized commercial Cu bactericide and fungicide; DuPont, Wilmington, DE) against a Cu-tolerant *X. perforans* strain, GEV485. The bacterial cells were grown on NA plates for 24 h at 28 °C, and bacterial cells were then transferred to NA plates containing Cu at 20 μg/mL; that is, Cu (II) sulfate pentahydrate (CuSO_4_·5H_2_O) (Sigma-Aldrich, St. Louis, MO, USA), and incubated for 24 h at 28 °C. Bacterial cells were collected from NA plates and suspended in sterile 0.01 M solution of MgSO_4_·7H_2_O (2.46 g/L) in deionized water. Suspensions were diluted to 10^5^ CFU/mL, and 20 μL of the bacterial suspension were transferred to 2 mL of each treatment in a sterile glass tube. Nano-MgO and Mg-formulated nanomaterials (SgMc, SgMg #3, and SgMg #2.5), were prepared at 100 and 1000 μg/mL. Kocide 3000 contained 30% metallic Cu in the form of copper hydroxide (Cu(OH)_2_). Kocide 3000 at 1 g/L contained Cu at approximately 300 μg/mL. Each treatment consisted of three replications for each bacterial strain. For the control group, the glass tube only contained 2 mL of sterile tap water and 20 μL of the bacterial working suspension. The tubes were incubated at 28 °C on an orbital shaker (150 rpm). At 15 min and 1, 4, and 24 h, 50 μL samples were extracted from each tube and plated on NA. NA plates were incubated at 28 °C for 48 h. Bacterial colonies were counted on each plate and converted to CFU/mL. The in vitro assay was repeated once.

### 3.5. Viability Assay Evaluating Bactericidal Activity

*X. perforans* strain GEV485 was used for the viability assay. Bacterial cells were incubated in nutrient broth at 28 °C on a shaker at 300 rpm for 16 h and harvested in the log phase. Bacterial cells were pelleted by centrifugation (5000 rpm for 10 min) and resuspended in 0.01 M MgSO_4_·7H_2_O, and the suspensions were adjusted to A_600_ = 0.3 (~5 × 10^8^ CFU/mL). Then, 500 μL of the bacterial suspension were transferred to 4.5 mL of Kocide^®^ 3000 (DuPont, Wilmington, DE, USA) at 1000 µg/mL. Sterilized tap water served as the control. The tubes were incubated at 28 °C on a shaker at 300 rpm for 4 h. After washing with 1 mL of 0.85% NaCl twice, 1 mL samples from each tube were stained using the LIVE/DEAD BacLight Bacterial Viability kit (L7007, Molecular Probes (Eugene, OR, USA), Invitrogen (Waltham, MA, USA)). The stain was a mixture of 1.5 mL Component A and 1.5 mL Component B. Following addition, the sample was incubated in darkness for 15 min at room temperature. Micrographs were taken with a Nikon Eclipse Ti inverted microscope (Nikon, Melville, NY, USA) at ×40 fluorescent optics using NIS-Elements imaging software (Ver. 3.0; Nikon). The dead cell/all cell ratio was calculated by ImageJ [34].

### 3.6. Greenhouse Experiments Evaluating Efficacy of Mg-Formulated Nanomaterials against Bacterial Spot Disease of Tomato

The following suspensions (200 mL each) were prepared in sterile tap water: nano-MgO- and Mg-formulated nanomaterials (SgMc, SgMg #3, and SgMg #2.5) at 100, 200, 500, and 1000 μg/mL, or a combination of Kocide 3000 (2.1 g/L) and Penncozeb 75DF (Cu-EBDC, 1.2 g/L; United Phosphorus, Inc., King of Prussia, PA, USA). Sterile tap water served as the control. Approximately 30 mL samples of the materials were sprayed on the foliage of 3- to 4-week-old Bonny Best tomato plants. The leaf surfaces which received the spray materials were allowed to air-dry before the leaf surfaces were sprayed with a suspension of the Cu-tolerant *X. perforans* strain, GEV485, adjusted to 5 × 10^8^ CFU/mL. The inoculated plants were then placed in plastic bags that were tightened around the base of the pot with a rubber band and placed in a growth chamber at 28 °C. After 48 h, the bags were removed and the plants were transferred to the greenhouse. The plants were assessed for disease severity and phytotoxicity using the Horsfall–Barratt disease severity scale [35] by rating every other day, beginning at 2 days post-inoculation, with the last rating occurring at 14 days post-inoculation. The disease rating assessed the overall affected area based on symptoms including lesions, foliar blighting, and discoloration. The area under the disease progress curve (AUDPC) was then calculated using the midpoint values [36]. There were four replications per treatment, and the experiment was repeated once.

### 3.7. Field Experiments Evaluating Efficacy of Mg-Formulated Nanomaterials against Bacterial Spot Disease of Tomato

Based on its performance in greenhouse experiments, nanomaterial SgMc was selected for field testing against bacterial spot disease of tomato in six trials (13 April to 15 June 2018, Quincy, FL; 20 April to 8 June 2018, Wimauma, FL;17 August to 4 October 2018, Quincy, FL; 7 September to 6 November 2018, Wimauma, FL; 18 April to 7 June 2019, Quincy, FL; and 3 April to 5 June 2019, Wimauma, FL).

Each treatment had 4 replications, consisting of 15 BHN 602 or Grand Marshall tomato plants in Quincy and HM1823 in Wimauma. The plots were arranged in a completely randomized block design. In Quincy, bed dimensions were 20.3 cm in height and 76.2 cm in width. Beds were spaced 1.8 m apart and plants were spaced 50.8 cm apart, within the row [37]. In Wimauma, beds were spaced 1.5 m apart and plants were spaced 60.96 cm apart within the row. Fertilizers were applied to plots based on soil type and cooperative extension recommendations [38]. Tomato plants were grown in the greenhouse in 128-cell containers before transplant. After transplanting, the treatments were sprayed on the foliar parts of the tomato plant at a rate of 1.2 L for 4 plots, 1 week prior to bacterial inoculation. The treatments consisted of formulated Mg nanomaterial SgMc or nano-MgO suspensions at 100 and 1000 μg/mL, with constant shaking while applying Kocide 3000 (2.1 g/L); the grower standard Kocide 3000 (2.1 g/L) in combination with Penncozeb 75DF (Cu-EBDC, 1.2 g/L); and an untreated water control. To ensure adequate disease development in the field plots, a suspension of the Cu-tolerant *X. perforans* GEV485 bacterial strain, adjusted to 5 × 10^8^ CFU/mL in deionized water, was applied to the foliage in the field by spraying the 1st, 8th, and 15th plants in each plot 1 week after the first chemical application. One liter of each antibacterial treatment was applied to each plot weekly using CO_2_ tanks and a u-shaped sprayer until one week before fruit harvesting. The plants were assessed for disease severity and phytotoxicity using the Horsfall–Barratt disease severity scale [35] every week after inoculation until harvest. The AUDPC was then calculated as described above [36]. There were four replications per treatment, and the experiment was conducted three times.

### 3.8. Statistical Analysis

The data collected from the in vitro assays and greenhouse and field experiments were evaluated for statistical significance using analysis of variance, followed by pairwise comparisons using either the least significant difference or the Student–Newman–Keuls method in IBM SPSS Statistics, version 22. A *p* value of 0.05 was used to evaluate significance.

## 4. Discussion

To minimize the inherent aggregation issues of MgO nanoparticles [20,32], three different formulated Mg nanomaterials (SgMc, SgMg #3, and SgMg #2.5) were prepared and studied. Upon exposure to humid conditions or water, the MgO surface is converted to Mg(OH)_2_ [20,21]. Therefore, an Mg(OH)_2_ nanomaterial (SgMc) was included in this study [27].

Considering the results of the DLS and SEM studies, formulating nano-MgO with citrate is beneficial for obtaining a smaller particle size that also exhibits improved colloidal stability. The adsorption of citrate on the surface of the particle was confirmed through FTIR and zeta potential measurements. It was noted that this change in surface chemistry (i.e., transition from the oxide/hydroxide to citrate, positive surface charge to negative surface charge) was expected to affect particle–bacteria interactions. Nonformulated nano-MgO has a positive charge on its surface, facilitating its interaction with the negatively charged bacterial membrane, while the formulated materials have a highly negative zeta potential, which can reduce the adherence of the particles to the bacterial membrane.

Most remarkably, XRD studies confirmed a complete transformation of MgO to Mg(OH)_2_ upon formulation with citrate. This transition must have occurred over time, as nano-MgO was not fully dissolved during the formulation process. Since the diffraction studies were performed a month after the formulation was prepared, the kinetics of this chemical transition remain unclear. The conversion of MgO to Mg(OH)_2_ in aqueous medium has been documented, but there is no published literature found to date documenting the antimicrobial properties of the end product.

Previous studies demonstrated that particulate size has a significant impact on the bactericidal activity of nano-MgO [11]. Similarly, Nakamura et al. (2021) recently demonstrated that Mg(OH)_2_ nanoparticles have higher antimicrobial potency against *E. coli*, suggesting that the contour or sharp edges of the particles can cause physical damage to the bacterial membrane, leading to bactericidal activity [39]. It is unclear whether formulating the particles would be beneficial or detrimental to their overall antimicrobial efficacy, considering the fact that SgMg materials are small and weakly crystalline, with a low probability of damaging bacterial membranes through physical means. Considering this, it is useful to compare the performance of formulated nano-MgO to that of Mg(OH)_2_ nanoparticles synthesized from soluble salts, and to assess the importance of crystallinity. Huang et al. (2018) utilized a co-precipitation method to prepare crystalline Mg(OH)_2_ nanoparticles stabilized with citrate ions [22]. This material was evaluated alongside the formulated nanomaterials to compare their performance and material properties in order to understand whether the crystallinity of the material played any role.

In the in vitro experiments (Figure 4 and Figure 5), both Cu-tolerant and Cu-sensitive *X. perforans* bacterial populations could not be recovered after exposure of the bacterial cells to SgMc at a concentration as low as 100 μg/mL for 15 min. In comparison, nonformulated nano-MgO showed bactericidal activity only after 4 h of exposure. However, SgMg #3 and SgMg #2.5, at 100 μg/mL, were only consistently bactericidal against Cu-sensitive *X. perforans*, despite having smaller particle sizes than nano-MgO. The bactericidal activity correlated with the high crystallinity present with nano-MgO and SgMc, while the inconsistent antimicrobial potency of SgMg #3 and SgMg #2.5 might have been related to the loss of crystallinity. Moreover, the viability assay (Figure 6) demonstrated the enhanced bactericidal activity of SgMc (100% mortality) against Cu-tolerant *X. perforans* at 100 μg/mL after 4 h, whereas the Cu bactericide only reduced recovery to about 80%, which was similar to the untreated water control.

To adequately protect plants against leaf infections, it is necessary to reduce the viability of phytopathogenic bacteria on the surface of treatment leaves. Our first greenhouse study (Figure 7) demonstrated a significant reduction in disease severity for all the formulated nanomaterials compared to the grower’s standard and untreated control. These results demonstrate the potential of formulated Mg nanomaterials to protect tomato against bacterial spot disease, as well as the limitations of Cu-EBDC in reducing disease severity. The second greenhouse study (Figure 8) showed a concentration-dependent reduction in AUPDC for all nanomaterials, including nonformulated nano-MgO. Interestingly, SgMg #3 and SgMg #2.5 did not significantly reduce disease severity compared to Cu bactericide when applied at 200 μg/mL and 100 μg/mL. Since there were a few months in between the second in vitro and greenhouse experiments, the stability of these two nanomaterials might have been compromised during storage. Therefore, future studies are needed to determine changes in the physical properties of the materials during storage conditions. Evaluating changes over time is important, given that a loss of crystallinity or an increase in particle size can affect the shelf life and antimicrobial activity of SgMg #3 and SgMg #2.5. Moreover, in plants, bacterial survival and infection is affected by leaf roughness [40], plant nutrition [41,42], chemical retention, and spatial distribution [43]. These factors often account for the differences in performance in plants compared to in vitro assays. Given its consistency in reducing disease, SgMc was selected for evaluation in the field conditions to manage bacterial spot disease in tomato plants.

Six field trials were conducted to assess the performance of SgMc in terms of protection against bacterial spot of tomato. The results demonstrated no consistent pattern to draw a definite conclusion as to the efficacy of SgMc in the field. SgMc, at concentrations as low as 100 μg/mL, was consistently effective in the greenhouse compared to the grower’s standard (Cu-EBDC), but it was not effective at reducing disease severity in the field at either location in the spring nor the fall of 2018 (Table 2 and Table 3). However, in 2019, both nano-MgO and SgMc reduced disease severity compared to the untreated control at both locations (Table 4). Comparing the performances of nano-MgO and SgMc in the greenhouse trials to those in the field trials, there was a noticeable reduction in efficacy. This difference was likely due to environmental variables that are not present in the greenhouse. Considering this, it appears that even though nano-MgO and SgMc possessed good bactericidal activity against the pathogen and provided disease protection in the greenhouse setting, they were significantly affected by unknown factors which reduced their effectiveness. Future studies are needed to determine which specific variable reduces the performance of these nanomaterials in the field compared to the greenhouse. Although SgMc was not consistent in reducing disease severity in the field, it still has potential to be an alternative to Cu in tomato transplant production against bacterial spot disease.

In conclusion, increasing the concentration of citrate used to formulate MgO nanoparticles reduces the particle size, induces changes in morphology, and reduces particle surface charge and crystallinity. These physical changes have a profound effect on the antimicrobial properties of the materials; formulations including SgMg #2.5 and SgMg #3, with higher concentrations of citrate, resulted in homogenized suspension compared to nonformulated nano-MgO materials. However, unlike nano-MgO, which was consistently lethal against Cu-tolerant *X. perforans* in former studies, the antibacterial activity of SgMg #2.5 and #3 was not consistent in vitro against the Cu-tolerant *X. perforans* strain. In this study, these two newly formulated materials, SgMg #2.5 and SgMg #3, were also compared to SgMc, which is a formulation of Mg(OH)_2_ nanoparticles synthesized from salt precursors due to their capabilities in bacterial disease management. As a result, the synthesized crystalline material SgMc exhibited more consistent antibacterial activity both in vitro and in the greenhouse compared to SgMg #2.5 and SgMg#3. In addition, this bactericidal activity correlated with the high crystallinity present in nano-MgO and SgMc, while the inconsistent antimicrobial potency of SgMg #3 and SgMg #2.5 might be related to loss of crystallinity. Future studies are needed to determine which specific variables reduce the performance of these nanomaterials in the field compared to under greenhouse conditions. Although SgMc did not provide significant disease severity reduction in the field, it still has the potential to be an alternative to Cu in tomato transplant production in the greenhouse against bacterial spot disease.

## Figures and Tables

**Table 1:** Characterization of the formulated MgO nanoparticles.

Name	Magnesium to Citrate mol Ratio	Average Hydrodynamic Diameter	PDI	Zeta Potential (mv)	pH at 1000 µg/mL
MgO	1.00 mol Mg:0.0 mol Citrate	2586	1	+11.4	10.20
SgMg #3	1.00 mol Mg:0.42 mol Citrate	2140	0.658	−43.3	10.72
SgMg #2.5	1.00 mol Mg:0.63 mol Citrate	88.8	0.360	−36.0	10.70
SgMc	1.00 mol Mg:0.50	279.8	0.476	−16.9	10.69

**Table 2:** Bacterial spot disease severity, as indicated by the area under disease progress curve (AUDPC), in tomato plants in the field treated with nano-MgO and SgMc in comparison to Cu-based bactericide (Kocide^®^ 3000), the grower standard (copper–mancozeb), and the untreated water control in Quincy and Wimauma, FL, in the spring of 2018.

		AUDPC ^y^ in Different Seasons ^w^	
Treatment	Rate (µg/mL)	Quincy, FL 2018 Spring	Treatment
Nano-MgO	1000	1010.0 a	Nano-MgO
Nano-MgO	100	791.9 a	Nano-MgO
SgMc	1000	883.8 a	SgMc
SgMc	100	1036.0 a	SgMc
Kocide 3000	2100	846.1 a	Kocide 3000
Cu-EBDC ^x^		873.5 a	Cu-EBDC ^x^
Water (Untreated)		822.0 a	1520.3 a

Numbers with different letters in the same column showed significant differences (*p* = 0.05) based on least significant difference (LSD) statistical analysis using the IBM^®^ SPSS^®^ program. ^y^ The area under disease progress curve (AUDPC) was calculated using the midpoint values obtained using the Horsfall–Barratt disease severity scale (Barratt and Horsfall 1945) (Campbell and Madden 1990). ^x^ Cu-EBDC is composed of Kocide 3000 (2100 µg/mL) and Penncozeb^®^ 75DF (1200 µg/mL). ^w^ The field trials were conducted in two locations.

**Table 3:** Bacterial spot disease severity as indicated by the area under disease progress curve (AUDPC) on tomato plants in field plots treated with nano-MgO and SgMc, in comparison to Cu-based bactericide (Kocide^®^ 3000), the grower standard (copper–mancozeb), and the untreated water control in Quincy and Wimauma, FL, in the fall of 2018.

		AUDPC ^y^ in Different Seasons ^w^	
Treatment	Rate (µg/mL)	Quincy, FL 2018 Fall	Wimauma, FL 2018 Fall
Nano-MgO	1000	510.4 a	585.9 ab
Nano-MgO	100	502.1 a	570.4 ab
SgMc	1000	602.6 a	437.3 a
SgMc	100	604.4 a	465.1 a
Kocide 3000	2100	592.1 a	743.3 b
Cu-EBDC ^x^		519.4 a	511.7 a
Water (Untreated)		596.8 a	583.7 ab

Numbers with different letters in the same column showed significant difference (*p* = 0.05) based on least significant difference (LSD) statistical analysis using the IBM^®^ SPSS^®^ program. ^y^ The area under disease progress curve (AUDPC) was calculated using the midpoint values obtained using the Horsfall–Barratt disease severity scale (Barratt and Horsfall 1945) (Campbell and Madden 1990). ^x^ Cu-EBDC is composed of Kocide 3000 (2100 µg/mL) and Penncozeb^®^ 75DF (1200 µg/mL). ^w^ The field trials were conducted in two locations.

**Table 4:** Bacterial spot disease severity as indicated by the area under disease progress curve (AUDPC) on tomato plants in the field, treated with nano-MgO and SgMc in comparison to Cu-based bactericide (Kocide^®^ 3000), the grower standard (copper–mancozeb), and the untreated water control in Quincy and Wimauma, FL, in the spring of 2019.

		AUDPC ^y^ in Different Seasons ^w^	
Treatment	Rate (µg/mL)	Quincy, FL 2019 Spring	Wimauma, FL 2019 Spring
Nano-MgO	1000	1424.6 ab	650.3 a
Nano-MgO	100	1119.6 a	691.3 ab
SgMc	1000	1274.5 ab	632.1 a
SgMc	100	1098.3 a	903.9 bc
Kocide 3000	2100	1481.9 ab	677.6 a
Cu-EBDC ^x^		1253.5 ab	870.3 b
Water (Untreated)		1657.1 bc	1033.2 bc

Numbers with different letters in the same column showed significant difference (*p* = 0.05) based on least significant difference (LSD) statistical analysis using the IBM^®^ SPSS^®^ program. ^y^ The area under disease progress curve (AUDPC) was calculated using the midpoint values obtained using the Horsfall–Barratt disease severity scale (Barratt and Horsfall 1945) (Campbell and Madden 1990). ^x^ Cu-EBDC is composed of Kocide 3000 (2100 µg/mL) and Penncozeb^®^ 75DF (1200 µg/mL). ^w^ The field trials were conducted in two locations.

## Data Availability

Data is contained within this article. Additional data can be made at request to corresponding authors.
